# Palaeontological Evidence for the Last Temporal Occurrence of the Ancient Western Amazonian River Outflow into the Caribbean

**DOI:** 10.1371/journal.pone.0076202

**Published:** 2013-09-30

**Authors:** Orangel Aguilera, John Lundberg, Jose Birindelli, Mark Sabaj Pérez, Carlos Jaramillo, Marcelo R. Sánchez-Villagra

**Affiliations:** 1 Instituto de Biologia, Programa de Pós-graduação em Biología Marinha, Universidade Federal Fluminense, Campus do Valonguinho, Niterói, Rio de Janeiro, Brasil; 2 Department of Ichthyology, The Academy of Natural Sciences, Philadelphia, Pennsylvania, United States of America; 3 Departamento de Biologia Animal e Vegetal, Universidade Estadual de Londrina, Londrina, Paraná, Brazil; 4 Smithsonian Tropical Research Institute, Balboa, Ancon, Panamá, Republic of Panama; 5 Palaeontological Institute and Museum, University of Zürich, Zürich, Switzerland; Team 'Evo-Devo of Vertebrate Dentition', France

## Abstract

Fossil catfishes from fluvio-lacustrine facies of late Miocene Urumaco, early Pliocene Castilletes and late Pliocene San Gregorio formations provide evidence of a hydrographic connection in what is today desert regions of northern Colombia and Venezuela. New discoveries and reevaluation of existing materials leads to the recognition of two new records of the pimelodid *Brachyplatystoma* cf. *vaillantii*, and of three distinct doradid taxa: *Doraops* sp., *Rhinodoras* sp., and an unidentified third form. The presence of fossil goliath long-whiskered catfishes and thorny catfishes are indicative of the persistence of a fluvial drainage system inflow into the South Caribbean during the Pliocene/Pleistocene boundary, complementary to the previous western Amazonian hydrographic system described from the Middle Miocene Villavieja Formation in central Colombia and Late Miocene Urumaco Formation in northwestern Venezuela. The Pliocene Castilletes and San Gregorio formations potentially represent the last lithostratigraphic units related with an ancient western Amazonian fish fauna and that drainage system in the Caribbean. Alternatively, it may preserve faunas from a smaller, peripheral river basin that was cut off earlier from the Amazon-Orinoco, today found in the Maracaibo basin and the Magdalena Rivers.

## Introduction

The Amazon and Orinoco rivers of South America are major reservoirs of aquatic biodiversity in their waters and in the drainages surrounding them [1]. Thus, reconstructing the history of those major river systems is paramount to understanding the origin and evolution of that biodiversity and its geographic history. The Miocene-Pliocene freshwater fossil fish records of northwestern South America serve to test biogeographic hypotheses about major hydrographic changes in the fluvial systems. Those changes have been hypothesized to result from the rise of the eastern Andean cordillera and Sierra de Perijá in Colombia, and the Mérida Andes and western coastal cordillera in Venezuela. The rise of the eastern cordillera in Colombia and the western coastal cordillera in Venezuela isolated the inflow of the northern drainage system of the Amazonian effluent to the Proto-Caribbean [2,3]. The resulting changes in the drainage system produced loss of habitat and regional faunal extinction, in what is today an arid zone along the South Caribbean coast. Here, we present new fossil evidence that provides an unequivocal test for these changes and new geographical and temporal information.

A large body of geological and paleontological evidence from the Miocene suggests the presence of a large river system that flowed from far south in western Amazonia into the Andean foreland basin, which then drained into the Caribbean in northern Venezuela (e.g. [2-11]), and the continuum of the Amazonian forest into northwestern Venezuela during the Miocene [12]. In contrast, the structural and stratigraphic data from outcrops and subsurface units from the Maracaibo Basin and other basins of northwestern Venezuela and northeastern Colombia [13-15] show no evidence for deltaic deposits that are expected with the outflow of a major river system. Contrary to the fossil evidence treated here, those authors concluded that during the Oligocene and Miocene, the drainage of northern South America was isolated from the main continental drainage by highlands underlain by the Lara Nappes. Consequently, the ancestral western Amazonian river drainage flowed eastward, well south of the Falcón Basin as early as the Lower Oligocene. Therefore, the connection between southeastern Colombian and the Venezuelan Caribbean via a large persistent ancestral river remains controversial. The study of fossil catfishes reveals information to address this issue.

Fossil siluriform catfishes from tropical South America comprise the freshwater families Pimelodidae (long-whiskered catfishes), Doradidae (thorny catfishes), Loricariidae (suckermouth armored catfishes) and Callichthyidae (armored catfishes). All of the fossil taxa identified to or below the genus level have congeneric extant representatives in the Neotropical ichthyofauna that are widely distributed in the Amazon, Orinoco and Essequibo rivers and their tributaries. The fossil record from the sedimentary units Middle Miocene La Victoria and Villavieja (Honda Group) in Colombia [16], Late Miocene Urumaco in Venezuela [17], Late Miocene Solimões/Pebas (Madre de Dios: Iñapari) in Brazil, and Early to Late Miocene Solimões/Pebas in the Upper Amazon Basin, Brazil, Colombia and Peru [8] preserve a large diversity of vertebrates, including freshwater catfishes (see [18]). Representatives of Pimelodidae include: † *Brachyplatystoma* *promagdalena* Lundberg 2005 [19] and B. cf. *vaillantii* Valenciennes 1840 [20], † *Phractocephalus* *nassi* Lundberg and Aguilera 2003 [9]; † *P* *. acreornatus* Aguilera et al. 2008 [21], and *Phractocephalus* sp. (in [18,21]); *Platysilurus* sp. (in [10]); *Zungaro* and fossils tentatively referred to *Pimelodus* [18]. The doradid fauna includes at least one fossil species, † *Dorasdioneae* Sabaj Pérez et al. 2007 [10], *Oxydoras* cf. *niger* [18], a cleithrum assigned to *Rhinodoras* cf. *thomersoni* Taphorn and Lilyestrom 1984 [10,18,22], several unidentified doradids [18], and two additional taxa treated herein. Additional catfishes include members of Callichthyidae, Loricariidae and Pseudopimelodidae [18]. Those fossils facilitate a paleobiogeographic interpretation of the ancient Amazon-Orinoco flow into the Caribbean Sea and the subsequent changes to that drainage system.

New and reinterpreted fossils of freshwater catfishes Pimelodidae and Doradidae, and the persistence of related modern taxa in the Maracaibo, Orinoco, Amazon and Paraná-Paraguay basins are the object of this study. Those discoveries provide a testimony of the hydrographic and climate change associated with the Pliocene/Pleistocene boundary in South America.

## Material and Methods

**Figure 1:**
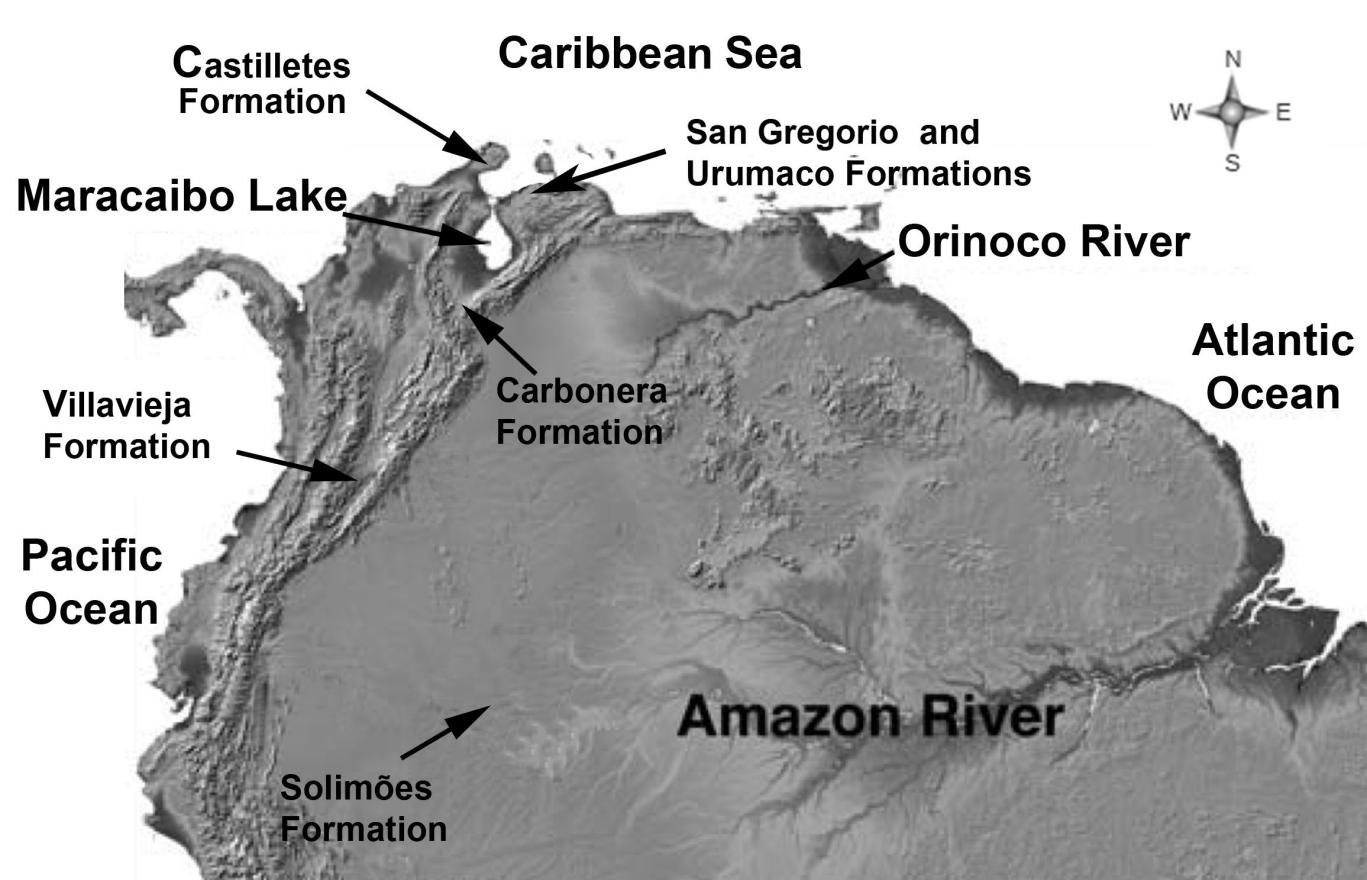
Map of northern South America. Marked are the locations of the outcrops containing fossil freshwater species that document the presence in Neogene times of a major drainage around what is today arid areas around the Lake Maracaibo (modified from Lundberg et al. 2010).

A fragmented neurocranium of *Brachyplatystoma* cf. *vaillantii* was collected from the upper member of the Urumaco Formation (Late Miocene), North Corralito, Urumaco trench, Venezuela (11°14’ 39. 3″ N; 70°16’25.7″ W). A single Weberian apparatus of B. cf. *vaillantii* was collected from the top of the Castilletes Formation (Early Pliocene), Cocinetas Basin, Alta Guajira Peninsula, Colombia (11°50’57.8″ N, 71°19’28.7″ W). One nearly complete pectoral spine identified as Doradidae and five broken spines were collected from the same outcrops directly on the surface (11°51’04.4″ N, 71°19’26.6″ W). Three doradid pectoral spine fragments were collected from the San Gregorio Formation, Vergel Member (Early Pliocene), East to San Gregorio, Falcón State, Venezuela (11°17’53.9″ N, 70°14’33.7″ W). In addition, new fossiliferous localities provide three news doradid skulls and one skull fragment from the Upper Member of the Urumaco Formation, North Corralito (11°14’44.7″ N, 70°16’26.4″ W) and from the Tío Gregorio locality (11°14’43.0″ N, 70°18’19.1″ W) (Figure 1).

All necessary permits were obtained for the described study, which complied with all relevant regulations. Specimen numbers are provided below, with complete repository information, including museum name and geographic location. Permission for fieldwork conducted by the Universidad Nacional Experimental Francisco de Miranda (Prof. Orangel Aguilera) was provided by the Instituto del Patrimonio Cultural and the Alcaldía Bolivariana del Municipio, Urumaco.

The sandstone matrix surrounding the specimens was removed with an electrical drill and by manual cleaning with fine tipped dental tools. The fossils were studied and illustrated using light stereomicroscopy. Comparative material included dry skeletons of Neotropical Pimelodidae and Doradidae deposited at the Museu Paraense Emilio Goeldi, Brazil (MPEG), Instituto de Pesquisas da Amazonia, Brazil (INPA), the Museu de Zoología da Universidade de São Paulo (MZUSP) and the Academy of Natural Sciences, Philadelphia, USA (ANSP). Nomenclature is based on Lundberg [19] for the pimelodid Weberian apparatus, and Gayet and Neer [23] for the pectoral spines. Measurements of doradid skulls are presented in Table 1.

**Table 1:** Doradid catfish skull measurements.

	***Doraops* sp.**	***Doraops* sp.**	***Doraops* sp.**	**Doradidae Genus & species indet. 1**	***Rhinodoras* sp.**	***Rhinodoras* sp.**
	**UNEFM-PF-0502**	**UNEFM-PF-0501**	**UNEFM-PF-0415**	**UNEFM-PF-0157**	**UNEFM-PF-0477**	**UNEFM-PF-0503**
**Maximum length**	133.8	125.4	117.1	102.2	103.4	120.5
**Maximum width**	78.6	54.5	55.2	80.1	39.4	49.3
**Maximum depth**	31.2	31.8	29.1	31.3	30.4	29.2
**Distance from posterior rim of fontanel to A P-S**	17.6	21.5	broken	broken	15.8	16.2
**Maximum length of P-S**	34.2	38.9 (broken)	36.1	35.0	32.7	31.5
**Maximum width of P-S at or approx. pitline**	26.2	27.8	26.3	22.4	22.2	24.9
**Maximum length of ANP at midline**	26.1	29.2	26.7	broken	21.6	28.3
**Maximum width of ANP**	22.8	broken	20.5	broken	broken	22.6
**Width of ANP along suture with supraoccipital**	17.2	broken	17.7	broken	19.5	16.7
**Length of anterolateral margin of ANP**	12.4	15.1	11.3	broken	11.0	10.2
**Length of posterolateral margin of ANP**	17.9	22.1	20.8	broken	19.4	20.0
**Middorsal length of MNP**	13.1	14.5	13.3	broken	broken	13.1
**Width of MNP at midlength of middorsal line**	35.4	broken	39.2	28.7	broken	33.2
**Length of suture ANP, MNP and E to LM**	19.9	broken	20.4	broken	broken	19.7
**Maximum width of basioccipital**	27.4	29.2	24.8	23.2	21.8	22.9

Abbreviations: AP-S anteriormost margin of parieto-supraoccipital; ANP anterior nuchal plate E epoccipital; LM lateral margin; MNP middle nuchal plate; NP nuchal plate; PS parieto-supraoccipital.

## Geological Setting

**Figure 2:**
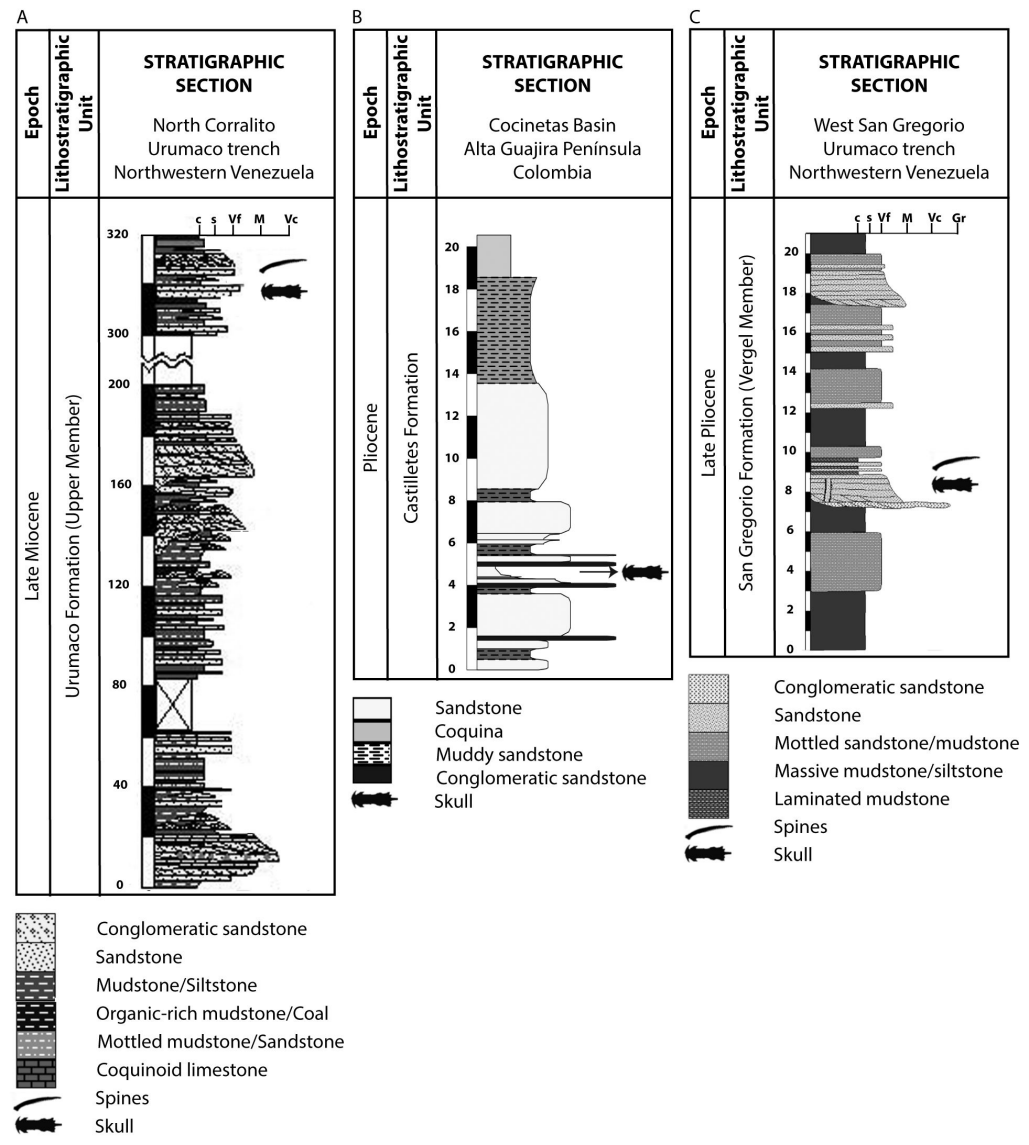
Stratigraphic sections. **A.** North Corralito, Urumaco trough, Venezuela (originally from Quiroz and Jaramillo 2010, modified from Aguilera and Marceniuk 2010); **B.** Cocinetas Basin, Alta Guajira Peninsula, Colombia (Carlos Jaramillo unpublished, section described by Federico Moreno and drawn using SDAR, a new stratigraphic software developed by STRI); C. East San Gregorio, Urumaco trough, Venezuela (Scheyer et al. 2013).

The stratigraphic section of the Late Miocene Urumaco Formation [24-26] in northwestern Venezuela includes three members comprising 2,560 m of sedimentary sequence. The upper member, where the fossils were collected (Figure 2A), comprises gray to brown, often limey claystone with thin intercalated and locally conchiferous sandstones. Several localities and levels have concentrations of vertebrate fossils. The vertebrate fauna includes marine, estuarine and freshwater fishes, terrestrial, freshwater and marine turtles and crocodilians, and terrestrial and aquatic/semiaquatic mammals (see 17).

The Castilletes Formation [27,28] outcrops in the Alta Guajira Peninsula [29], northern Colombia, is characterized by marly limestones, clays, calcareous and non-calcareous sandstones, and conglomerates. Toward the base of the formation, the limestones are coarse textured, marly, argillaceous, sandy fossiliferous, and fairly indurate (Figure 2B). The clays are silty, brown to buff, gray, greenish-gray and reddish, with some sandy zones [28]. Towards the top, where the fossils were collected, the reddish-yellow sandstones and conglomerates sandstones are more common, poorly sorted and with matrix supported in channel lenses. The unit rests conformably on the Jimol Formation and the upper contact is not exposed. The Castilletes Formation is ~340 m thick in the study area. The unit was deposited in a very shallow marine environment [28] intermixed with continental facies produced by fan delta progradation and fluvial deposits. The Castilletes Formation is rich both in marine and terrestrial fossils including plants, mammals, crocodiles, turtles, bivalves, gastropods, crabs and fishes. Dating of the lower Castilletes Formation is still controversial. Renz [27] suggested an Early Miocene age for the basal sediments based on the foraminiferan *Miogypsina* *antillea* Cushman 1919 [30]. Bürgl [31] estimated a Middle Miocene age, and Rollins [28] proposed Miocene to Early Pliocene based upon its stratigraphic position. The Weberian apparatus and pectoral spines were found near the top of the Castilletes Formation and the section is about 6 m thick. The upper Castilletes are stratigraphically equivalent to the Farrallones/Cerro Castilletes locality in the Cocinetas basin and dated as Early Pliocene (3.2 to 1.7 Ma: C. Jaramillo, pers. com.).

The San Gregorio Formation [25,32] is exposed in the north-central area of the Falcón State coastal plain, 10 km north of the town of Urumaco, east of San Gregorio, northwestern Venezuela. The Vergel member, where the fossils were collected, is the lowest of three members of the San Gregorio Formation (Figure 2C), and it is composed of approximately 85% limestones, 5% sandstones, and 10% conglomerates, encompassing 350 m at the type section [33,26], which overlies the Late Miocene to Early Pliocene Codore Formation. Smith et al. [34] suggested Middle to Late Pliocene based on foraminifera, and Vucetich et al. [35] suggested Late Pliocene based on rodents. The paleoenvironmental interpretation corresponds to a distal alluvial fan, crossed by low-sinousity channels. The lateral outflow of these channels during an inundation event form sub-aerial savannas, such as wetlands. Those inundated soils resulted in a paleosoil over sandy deposits, characterized by the presence of a terrestrial and semi-aquatic fossil assemblage, including mammals and crocodiles [35,36].

### Systematic Paleontology

Siluriformes *sensu* Berg 1940 [37]
Pimelodidae *sensu* Lundberg and Littmann 2003 [38]
Brachyplatystomatini Lundberg and Akama 2005[39]

*Brachyplatystoma* Bleeker 1862[40]

**Figure 3:**
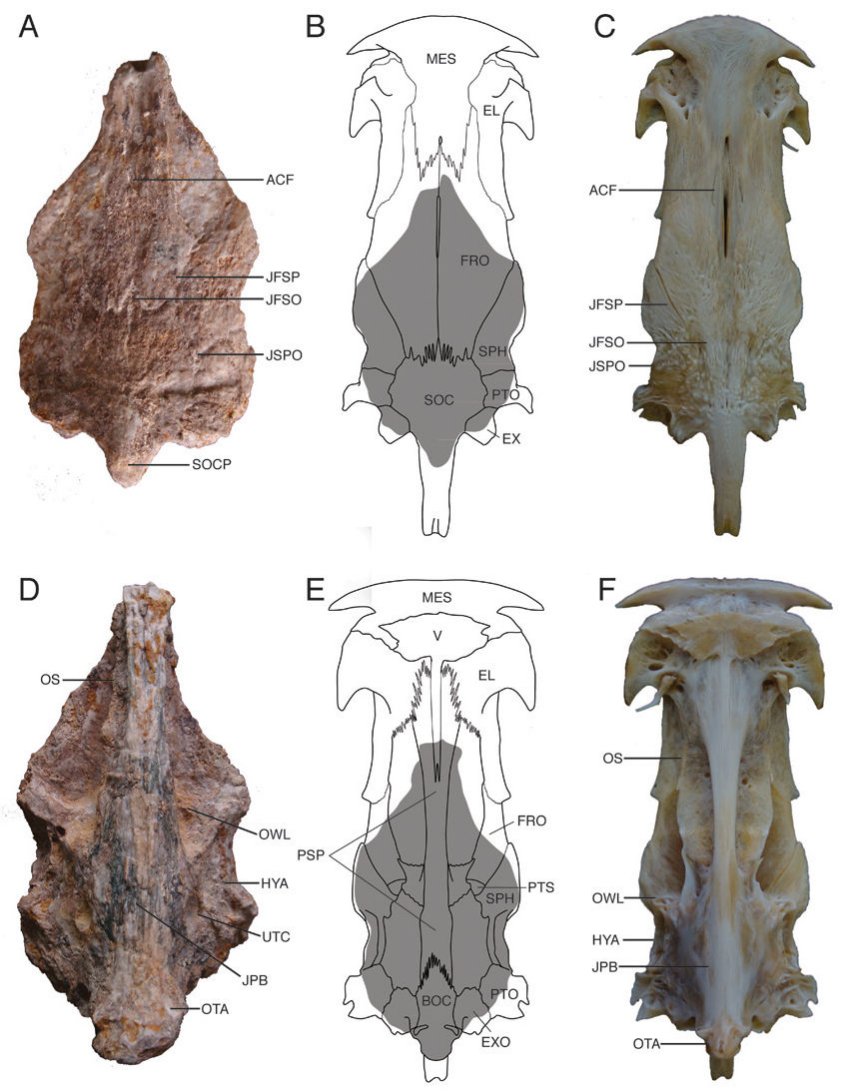
Neurocrania of fossil and modern *Brachyplatystoma* . **A**-**C**. Dorsal views, A. fossil † *Brachyplatystoma* cf. *vaillantii*, UNEFM-PF-500; B. line drawing of modern *B* *. vaillantii* with superimposed shaded approximation of fossil specimen, C. modern *B* *. vaillantii* . **D**-**F**. Ventral views of same. Abbreviations: **ACF** – anterior cranial fontanelle; **BOC** – basioccipital; **EL** – lateral ethmoid; **EX** – extrascapular; **EXO** – exoccipital; **FRO** – frontal; **HYA** – hyomandibular bone articulation site; **JFSO** – frontal-supraoccipital joint; **JFSP** – frontal-sphenotic joint; **JPB** – parasphenoid-basioccipital joint; **JSPO** – sphenotic-pterotic joint; **MES** – mesethmoid; **OS** – orbitosphenoid; **OTA** – articulation site of ossified transscapular ligament; **OWL** – otic capsule anterior wall; **PSP** – parasphenoid; **PTO** – pterotic; **PTS** – pterosphenoid; **SPH** – sphenotic; **SOC** – supraoccipital; **SOCP** – supraoccipital process; **UTC** – utricular chamber.

*Brachyplatystoma* cf. *B* *. vaillantii* Valenciennes 1840[20]
Figure 3A, 3D., 4 A-B, 4D.
b. cf. *vaillantii* in Lundberg [41]: 76-78 (figs. a-d, f).

**Figure 4:**
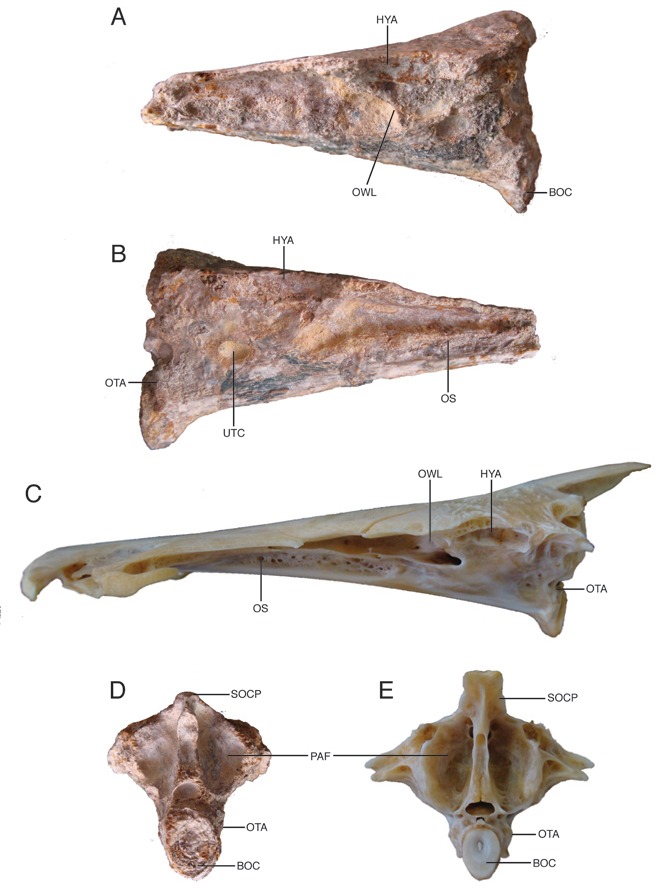
Neurocrania of fossil and modern *Brachyplatystoma* . **A**, **B**. Left and right lateral views of fossil † *Brachyplatystoma* cf. *vaillantii*, UNEFM-PF-500; C. Left lateral view of modern *B* *. vaillantii* . **D**. Occipital view of fossil † *Brachyplatystoma* cf. *vaillantii*, **E**. Occipital view of modern *B* *. vaillantii* . Abbreviations: **BOC** – basioccipital; **HYA** – hyomandibular bone articulation site; **OS** – orbitosphenoid; **OTA** – articulation site of ossified transscapular ligament; **OWL** – otic capsule anterior wall; **PAF** – paraoccipital fossa; **UTC** – utricular chamber; **SOCP** – supraoccipital process.

#### Newly examined specimens

**Figure 5:**
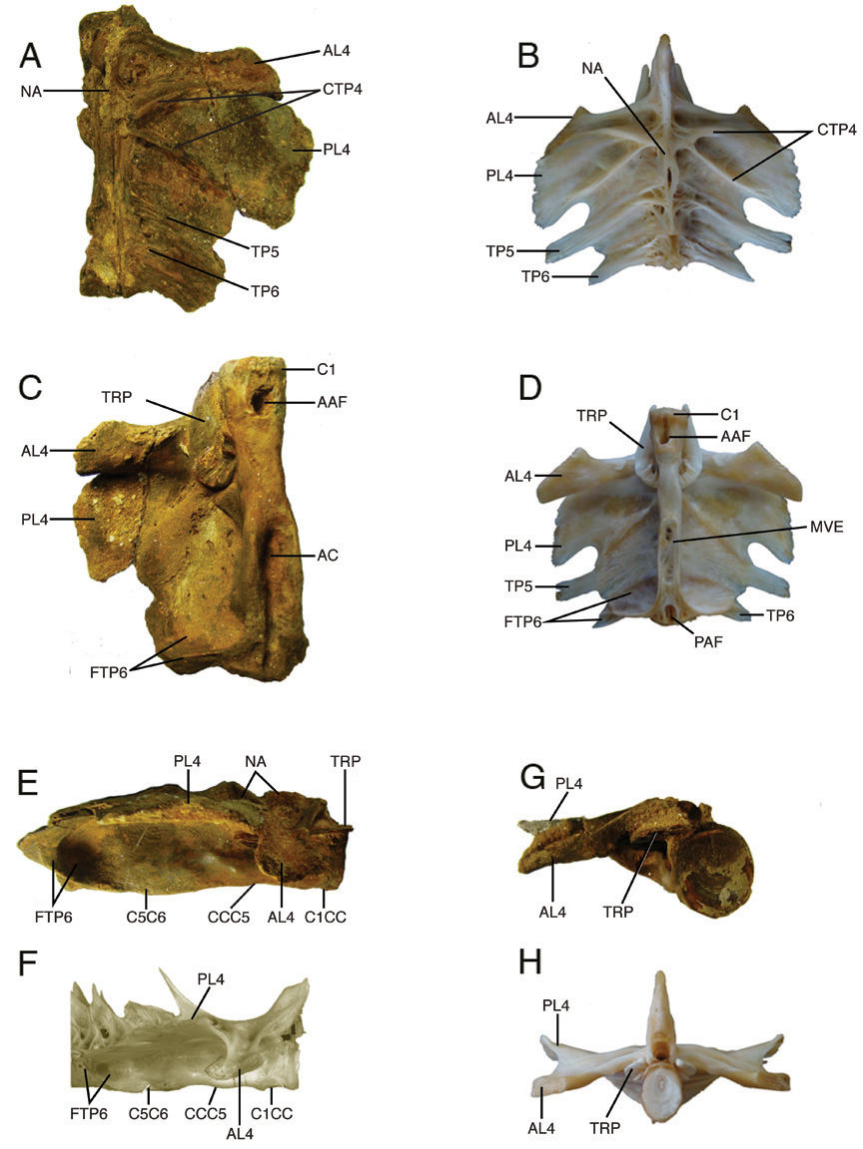
Weberian apparatus of fossil and modern *Brachyplatystoma* . Dorsal views of **A**. fossil † *Brachyplatystoma* cf. *vaillantii*, STRI-dbid 12959; **B**. modern *B* *. vaillantii* . **C**, **D**. Ventral views of same; **E**, **F**. right lateral views of same, **G, H**. anterior views of same. Abbreviations: **AAF** – aortic canal anterior foramen; **AC** - aortic canal; **AL4** – anterior limb of fourth transverse process; **CCC5** – compound centrum- fifth centrum joint; **C1** - first centrum; **C1CC** - first centrum-compound centrum joint; **C5C6** - fifth centrum- sixth centrum joint; **CTP4** - crests on fourth transverse process; **FTP6** – fossa and vertical wall of sixth transverse process; **NA** – neural arch; **PAF** – aortic canal posterior foramen; **PL4** - posterior limb of fourth transverse process; **TP5** - fifth transverse process; **TP6** - sixth transverse process; **TRP** – tripus.

Neurocranium without anterior ethmoid/vomerine region, UNEFM-PF-500 (Figures 3A, 3D, 4 A-B, 4D), from the Urumaco Formation (Upper Member), Late Miocene, North Corralito, Urumaco trough, Northwestern Venezuela (11°14’ 39. 3″ N; 70°16’25.7″ W), and Weberian apparatus, STRI-dbid 12959 (Figures 5A, 5C, 5E, 5G), from the Upper Castilletes Formation (top), Early Pliocene, Cocinetas Basin, Alta Guajira Peninsula (11°50’57.8″ N, 71°19’28.7″ W), northern Colombia.

#### Description

The fossil neurocranium (Figures 3A, 3D, 4 A-B, 4D) closely matches modern *B* *. vaillantii* (Figures 3C, 3F, 4C, 4E) in shape and anatomical details, including species-diagnostic features of the sphenoid, otic and occipital regions. Other parts of the fossil skull are severely abraded or missing (Figure 3B, 3E).

In dorsal view the skull roof (Figure 3A-B), including frontals, sphenotics, pterotics, extrascapulars and supraoccipital, is mostly flat except for the distinctly humpbacked base of the supraoccipital posterior process and the frontals are shallowly concave before the frontal-supraoccipital suture. The skull roofing bones articulate with shallow to deeply interdigitating joints. Bone surface texture varies from nearly smooth anteriorly to coarsely striate and somewhat tuberculate over the middle to posterior elements. The anterior cranial fontanelle on the midline between the frontals is narrowly open and terminates before the frontal-supraoccipital joint; a shallowing midline trough crosses that joint; the posterior cranial fontanelle is closed. The frontal-sphenotic joint runs anterolaterally and nearly straight from its junction with the supraoccipital to the skull margin posterior to the orbit and anterior to a laterally convex bulge of the sphenotic. Posterior to the convex lateral bulge, the edge of the sphenotic and pterotic traces a characteristic concave curve. The body of the pterotic is subquadrangular; its posterolateral corner is broken away and abraded. The sphenotic-pterotic joint runs medioposteriorly from the concave skull margin to the supraoccipital. The extrascapular, wedged between the supraoccipital and pterotic, is subtriangular, relatively large and projecting beyond the otherwise concave occipital margin. The base of the supraoccipital posterior process is about as wide as each adjacent extrascapular; the posterior process itself is broken away close to the base. The supraoccipital-frontal joint is transverse and coarsely serrate, and its joints with the sphenotic, pterotic and extrascapular curve gently along the convex sides of the supraoccipital body.

The ventral side of the neurocranium (Figure 3D-E) is dominated by the heavy midventral parasphenoid stem and basioccipital. The parasphenoid stem narrowly flares anteriorly to its broken anterior limit below the orbitosphenoid; the vomer is not preserved. The parasphenoid broadens posteriorly as the braincase floor and sidewalls, and posteriorly sutures deeply with the basioccipital. The parasphenoid joints with the pterosphenoids, sphenotics, prootics and exoccipitals are indistinctly preserved. The orbitosphenoid broadly contacts the frontals dorsally and the parasphenoid ventrally, its sidewalls are relatively deep, and bear worn remnants of horizontal shelves.

In ventral and lateral views (Figures 3D-E, 4A-B) the sphenotic and pterosphenoid are sharply elevated transversely to form the anterior wall of the otic capsule. The trigeminofacial nerve foramen is located ventral to the otic capsule wall, above the parasphenoid and anterior to the prootic. Ventral to the skull roof margin the sphenotic and pterotic form the laterally-concave and elongate articular surface for the neurocranial head of hyomandibular that begins on posterior end of the sphenotic and terminates on anterior end of the pterotic. The sidewall of the prootic is broken revealing the ovoid utricular chamber that contained the lapillus otolith. The basioccipital is exceptionally expanded ventrally and posteriorly to form a deeply ovoid and flat-faced occipital condyle. The basioccipital and adjacent exoccipital have the coarse attachment surface for the ossified transscapular ligament. The posterolateral sidewall of each exoccipital contains a prominent, circular vagal nerve foramen.

In posterior view (Figure 4D) the neurocranium shows the dorsally arched supraoccipital, strong median keel below the supraoccipital posterior process, foramen magnum, and prominent occipital condyle; laterally the paired, paroccipital fossae are tall and deeply concave. The dorsolateral pterotic corners of the occiput are not preserved.

The partial Weberian vertebral complex (Figures 5A, 5C, 5E, 5G) is three-dimensional, undistorted, although abraded, and includes parts of the anterior six vertebrae of which the second through fourth are fused as the Weberian compound vertebra [42]. The right tripus is present and articulated ventroanteriorly to the compound centra; the smaller Weberian ossicles are missing. The fossil Weberian complex closely matches modern *B* *. vaillantii* (Figures 5B, 5D, 5F, 5H) in shape and anatomical details including species-diagnostic features.

The Weberian complex bone texture is densely laminar unlike the cancellous or trabeculated texture seen in other species of *Brachyplatystoma* [19]. The first, compound, fifth and sixth vertebrae are enlarged and tightly jointed together although the interdigitating joints are mostly indistinct due to abrasion. The first vertebra comprises a centrum only without neural arches or transverse processes. This centrum is cylindrical, smooth-sided without pits or ridges and a little wider than the compound centrum. The anterior, articulation face of the first centum is slightly concave, ovoid in form and a little compressed laterally; the central focus of growth rings on the anterior face of this centrum is located above the center of the centrum at about one third of the depth below the dorsal rim.

In dorsal view (Figure 5A) the bases of otherwise broken neural arches flank the exposed neural canal across the compound, fifth and sixth vertebrae; anteriorly neural arch material of the compound centrum projects above the first centrum. Most of the much-enlarged anterior and posterior limbs of the right fourth transverse process and proximal parts of the right fifth and sixth transverse processes are preserved. In life these transverse processes form a bony sheet over the gas bladder. The anterior limb of the fourth transverse process is thick, with an elevated crest running along its anterior margin, and its tip is expanded and obliquely truncated anterolaterally across its surface of articulation with the posttemporo-supracleithrum. The posterior limb of the fourth transverse process is distinctively expanded, longer than the anterior limb, and has two dorsal crests forming a V-shaped pattern that is characteristic of *B* *. vaillantii* (Figure 5B); its lateral margin is incompletely preserved. The transverse processes of the fifth and sixth vertebrae are dorsally thickened along their central axes, but are broken distally so that their marginal shapes are indeterminate.

The centrum of the compound vertebra is about 3 times the ventrolateral length of the first centrum, anteriorly broadened at its joint with the first centrum and contains the large anterior foramen for passage of the dorsal aorta into the aortic canal deeply-embedded within the compound, fifth and sixth centra. However, in the fossil the bony floor of the aortic canal below sixth centrum is eroded off revealing the lumen of the aortic canal. Likely due to abrasion, there is no evidence of a coarsely textured midventral expansion (gas bladder attachment site) on the compound or fifth centra (compare Figure 5C-D)

The lateral surfaces of the compound, fifth and sixth centra (Figure 5E) and the ventral surfaces of the posterior limb of the fourth, fifth and most of the sixth transverse processes are covered with a smooth veneer of superficial ossification. Posteriorly, the sixth transverse process is much expanded ventrally and laterally to form an enlarged, bowl-shaped fossa that in life covers the posterior end of the gas bladder. This form of the sixth transverse process is characteristic of *B* *. vaillantii* (Figures 5B, 5D, 5F, 5H).

The right tripus Weberian ossicle (Figure 5C) and its associated os suspensorium are preserved in position on the sides of the first and compound centra and ventral to the anterior limb of the fourth transverse process. The tripus has the normal crescentic shape and striate posterior half where a suspensory ligament attaches.

#### Measurements

UNEFM-PF-504, Neurocranium; STRI-dbid 12959, Weberian apparatus length: 84.6 mm; preserved width: 58.0 mm.

Doradidae *sensu* Sabaj and Ferraris, 2003[43]
*Doraops* Schultz 1944[44]

**Figure 6:**
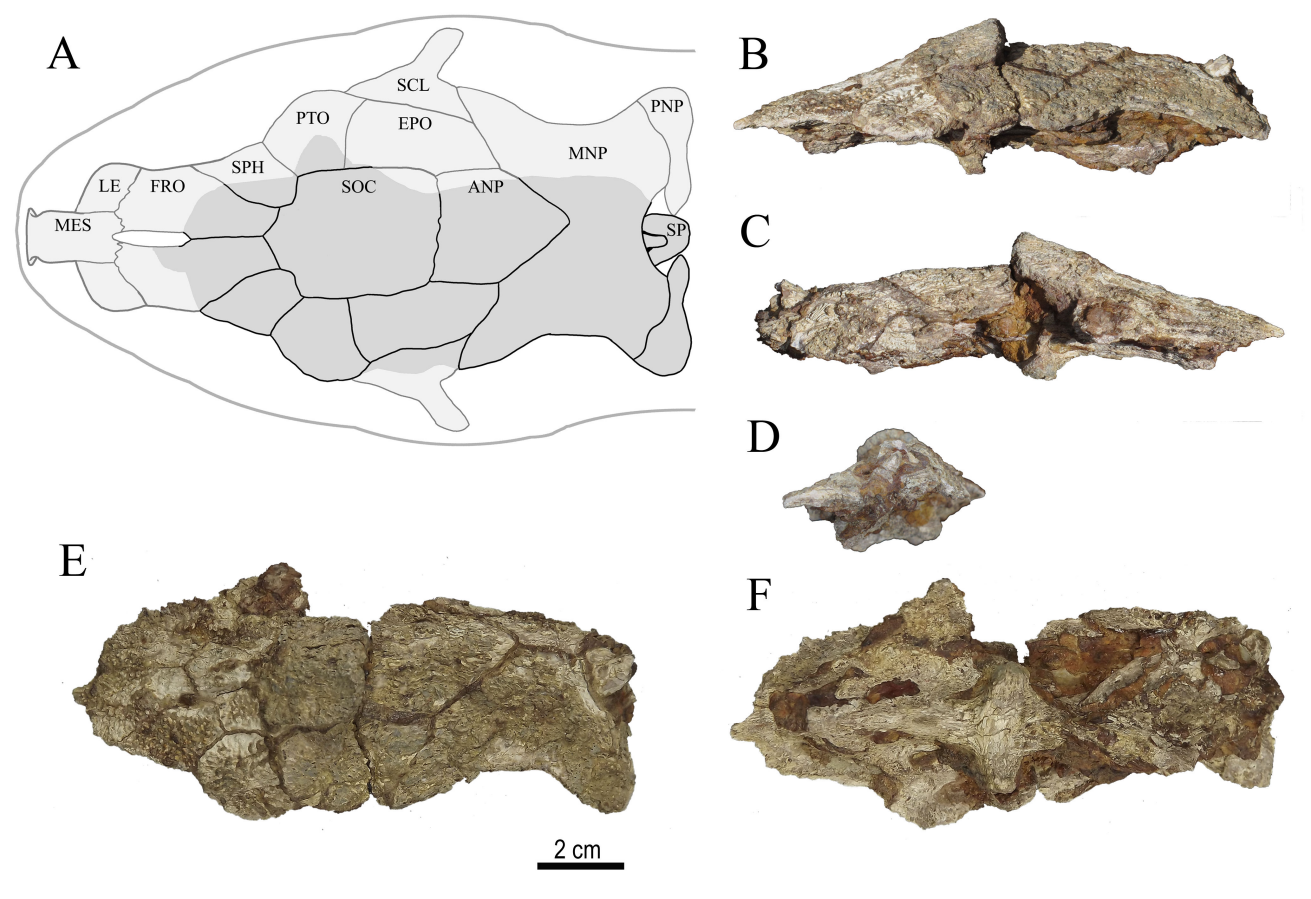
Neurocranium of *Doraops* sp., UNEFM-PF-501, from the Urumaco Formation, Upper Member, Late Miocene. North Corralito, northwestern Venezuela (11º 14’ 44.7” N, 70º 16’ 26.4” W). **A** schematic reconstruction and bones sutures (light gray: skull reference, dark gray: fossil skull); **B** lateral left view; **C** lateral right view; **D** posterior view; **E** dorsal view; **F** ventral view. Abbreviations: **ANP** – anterior nuchal plate; **EPO** – epioccipital; **FRO** – frontal; **LE** – lateral ethmoid; **MES** – mesethmoid; **MNP** – middle nuchal plate; **PNP** – posterior nuchal plate; **PTO** – pterotic; **SCL** – supracleitrum; **SOC** – supraoccipital; **SP** – dorsal-locking spine; **SPH** – sphenotic.

**Figure 7:**
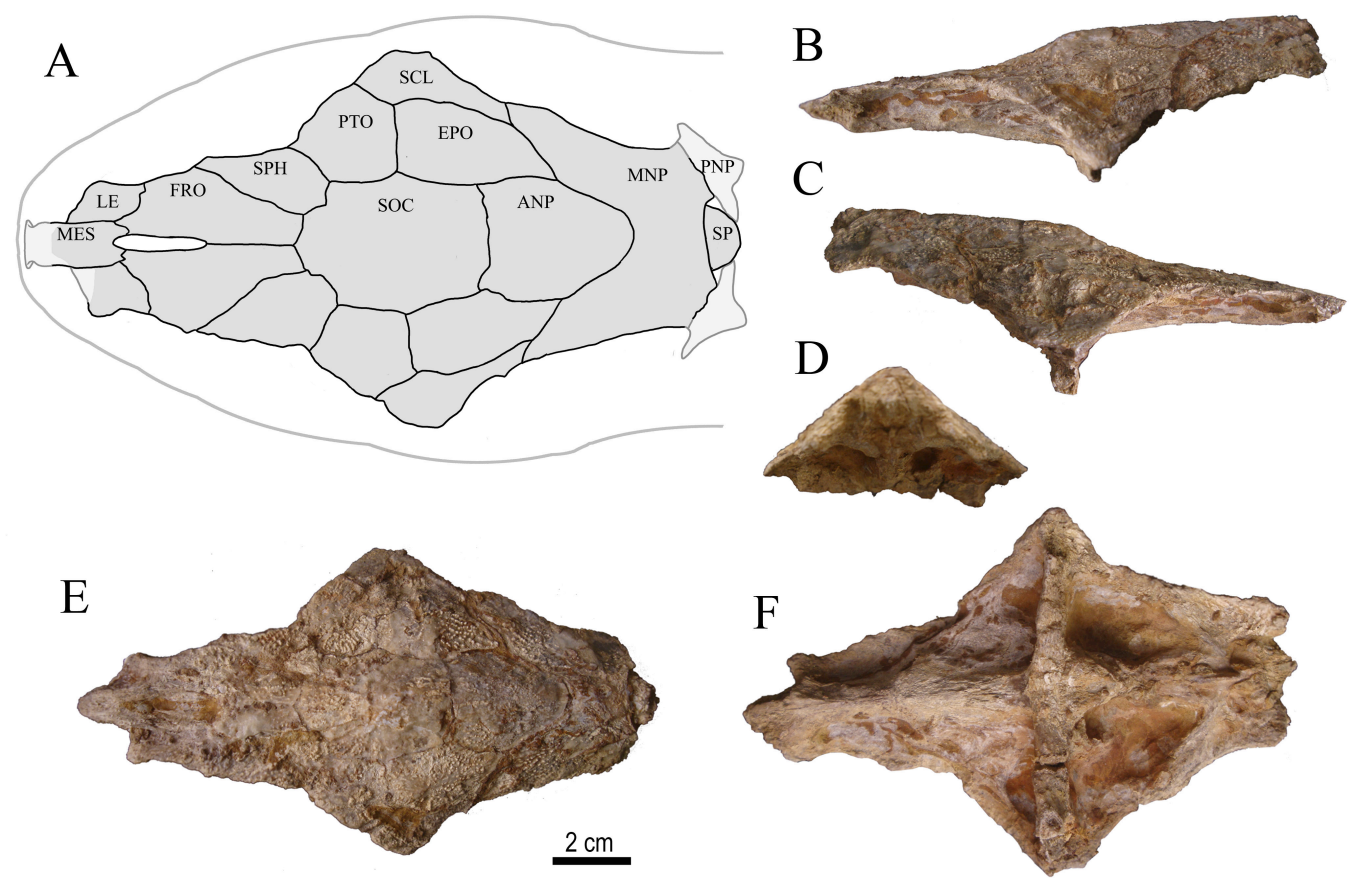
Neurocranium of *Doraops* sp., UNEFM-PF-502, from the Urumaco Formation, Upper Member, Late Miocene. Tío Gregorio, northwestern Venezuela (11º 14’ 43.0” N, 70º 18’ 19.1” W). **A** schematic reconstruction and bones sutures (light gray: skull reference, dark gray: fossil skull); **B** lateral left view; **C** lateral right view; **D** posterior view; **E** dorsal view; **F** ventral view. Abbreviations: **ANP** – anterior nuchal plate; **EPO** – epioccipital; **FRO** – frontal; **LE** – lateral ethmoid; **MES** – mesethmoid; **MNP** – middle nuchal plate; **PNP** – posterior nuchal plate; **PTO** – pterotic; **SCL** – supracleitrum; **SOC** – supraoccipital; **SP** – dorsal-locking spine; **SPH** – sphenotic.

*Doraops* sp.
Figures 6 A-F and 7 A-F; Table 1

cf. *Rhinodoras* sp. in Aguilera 2004 [45]: 63 (description, biogeography, figure of UNEFM-PF-0278).

Doradidae gen. et sp. indet. 1 in Sánchez-Villagra and Aguilera 2006 [46]: 215 (Table 1), 217 (Figure 2f of UNEFM-PF-0278).

**Figure 8:**
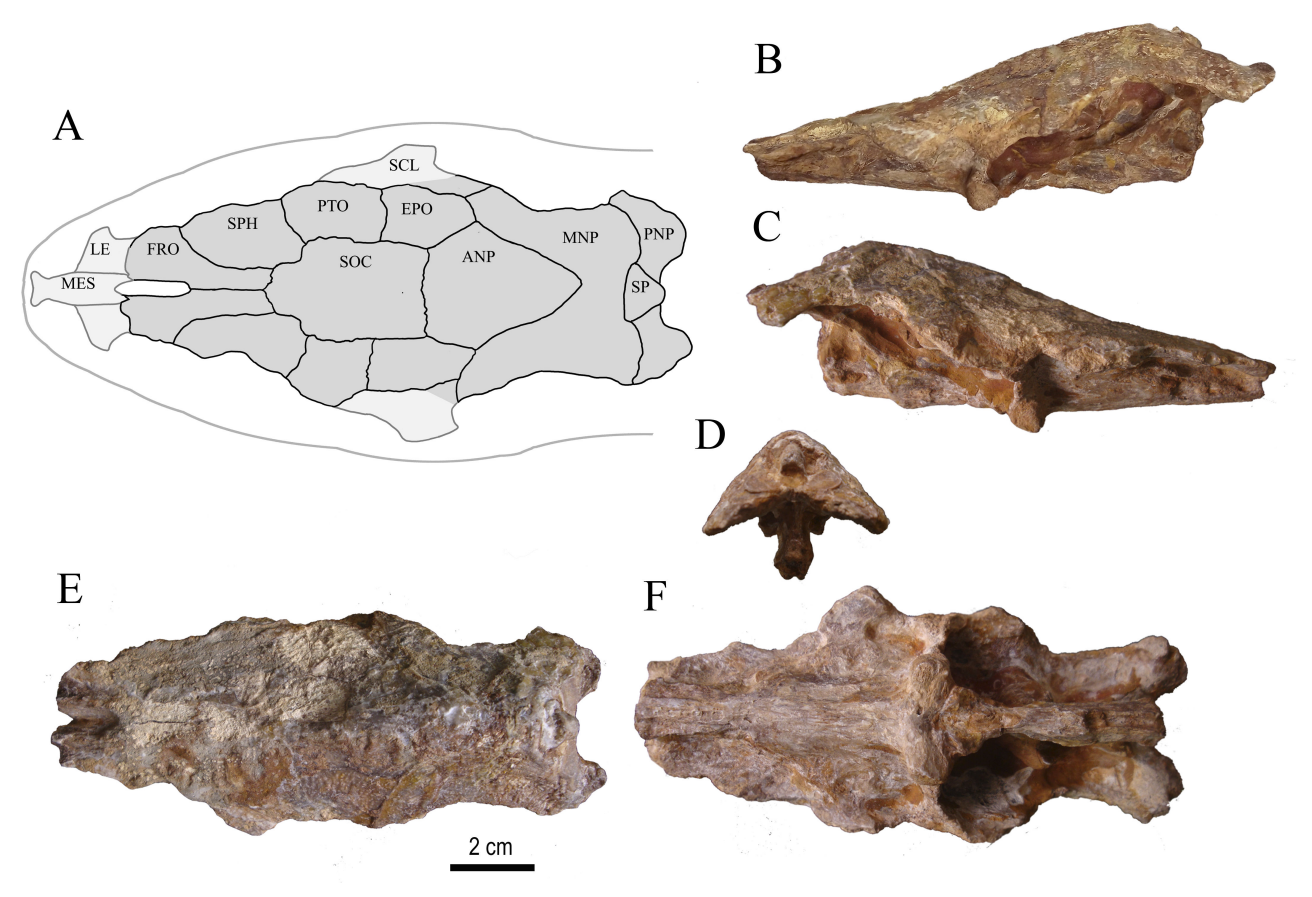
Neurocranium of *Rhinodoras* sp., UNEFM-PF-503, from Urumaco Formation, Upper Member, Late Miocene. North Corralito, northwestern Venezuela (11°14’44.7″ N, 70°16’26.4″ w). **A** schematic reconstruction and bones sutures (light gray: skull reference, dark gray: fossil skull); **B** lateral left view; **C** lateral right view; **D** posterior view; **E** dorsal view; **F** ventral view. Abbreviations: **ANP** – anterior nuchal plate; **EPO** – epioccipital; **FRO** – frontal; **LE** – lateral ethmoid; **MES** – mesethmoid; **MNP** – middle nuchal plate; **PNP** – posterior nuchal plate; **PTO** – pterotic; **SCL** – supracleitrum; **SOC** – supraoccipital; **SP** – dorsal-locking spine; **SPH** – sphenotic.

**Figure 9:**
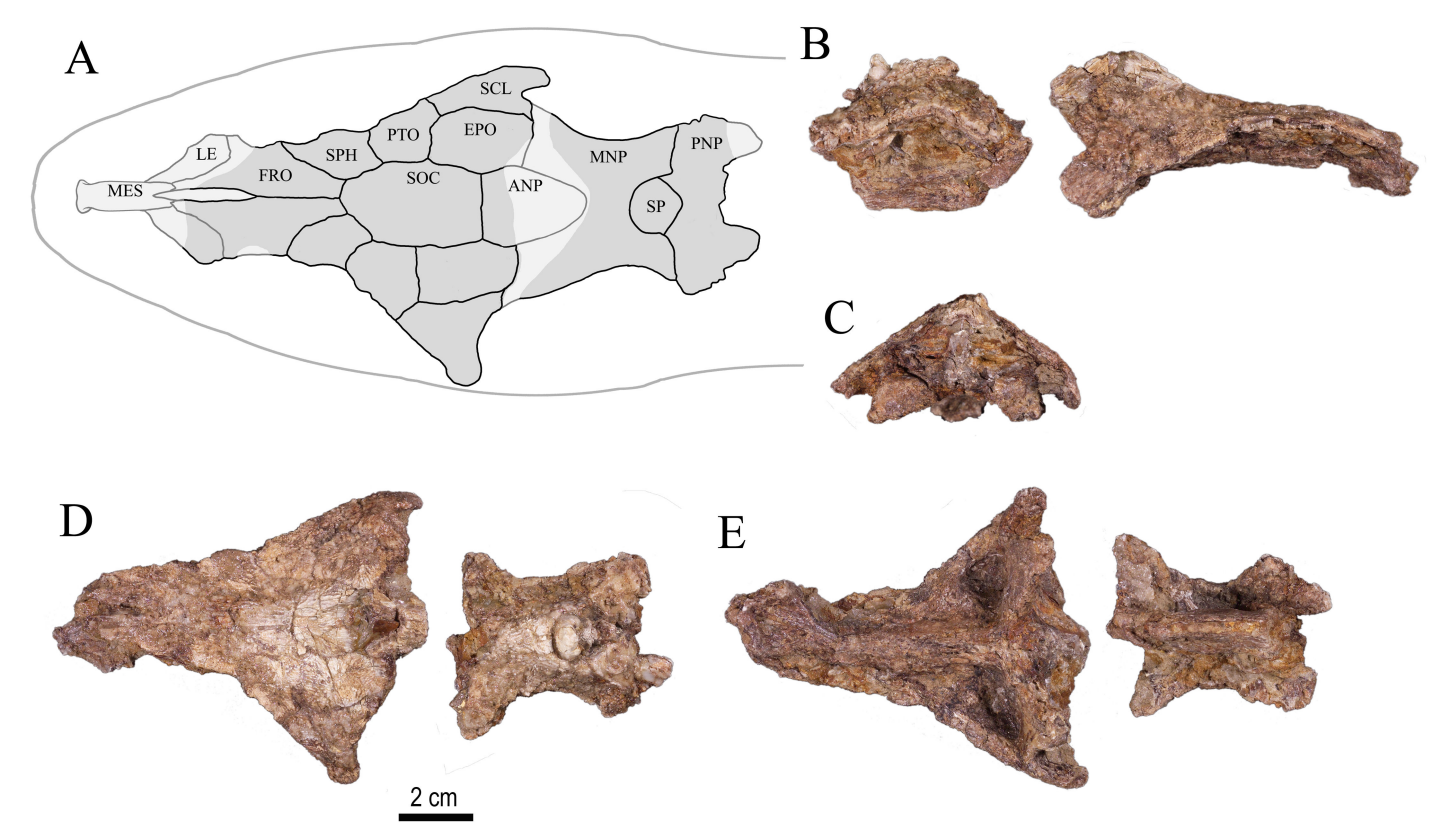
Neurocranium of Doradidae genus and species indeterminate 1, UNEFM-PF-0157, from Urumaco Formation. Las Huertas, northwestern Venezuela (11°14’ 00” N, 70°17’10″ W). **A** schematic reconstruction and bones sutures (light gray: skull reference, dark gray: fossil skull); **B** lateral view; **C** posterior view; **D** dorsal view; **E** ventral view. Abbreviations: **ANP** – anterior nuchal plate; **EPO** – epioccipital; **FRO** – frontal; **LE** – lateral ethmoid; **MES** – mesethmoid; **MNP** – middle nuchal plate; **PNP** – posterior nuchal plate; **PTO** – pterotic; **SCL** – supracleitrum; **SOC** – supraoccipital; **SP** – dorsal-locking spine; **SPH** – sphenotic.

*Doraops* cf. *zuloagai* Schultz, 1944 [44] in Sabaj Pérez et al. 2007 [10]: 167–174 (Figures 6, 8a, 9a–d), 194 (Appendix 3) (fossils from Urumaco Formation, Tío Gregorio, Falcón State, Venezuela: UNEFM-PF-0271, nearly complete neurocranium, nuchal shield, anterior vertebrae and dorsal-fin base; two major moieties separated across anterior nuchal plate and epioccipitals; UNEFM-PF-0415, partial neurocranium and nuchal shield; UNEFM-PF-0278, partial left pectoral girdle with articulated pectoral spine [spine incomplete, approximately distal half missing]; UNEFM-PF-0413, partial left pectoral girdle with articulated pectoral spine [spine incomplete, approximately distal third missing]). Lundberg et al. 2010 [18]: 293–294 (biogeography, comparisons to extant congener), 287 (Table 17.2).

#### Newly examined specimens

Neurocranium without anterior ethmoid/vomerine region, UNEFM-PF-501, from the Urumaco Formation, Upper Member, Late Miocene; North Corralito, northwestern Venezuela (11°14’44.7″ N, 70°16’26.4″ W); neurocranium without anterior ethmoid/vomerine region, anteriormost preserved bone frontal, left side with sphenotic, pterotic and epioccipital complete or nearly so; posttemporal-supracleithrum lacking; right side with small portions of sphenotic and pterotic; epioccipital and posttemporal-supracleithrum lacking; anterolateral border of anterior nuchal plate, and right halves of middle and posterior nuchal plates also lacking; spinelet preserved. UNEFM-PF-502, from the Urumaco Formation, Upper Member, Late Miocene, Tío Gregorio, northwestern Venezuela (11°14’43.0″ N, 70°18’19.1″ W); neurocranium nearly complete, lacking only anterior tip of mesethmoid, anterior portion of left lateral ethmoid, and ventralmost portion of both posttemporal-supracleithra; posterior nuchal plate almost completely lacking.

#### Description

In lateral view, dorsal profile of neurocranium nearly straight, except for gentle (UNEFM-PF-501) to pronounced (UNEFM-PF-502) inflection at midline of parieto-supraoccipital elevating nuchal region (Figures 6 and 7). In transverse plane, dorsal surface flattened anteriorly across frontals, gently arched across pterotics and anterior half of parieto-supraoccipital, progressively more arched posteriorly, reaching an angle of approximately 120° (UNEFM-PF-501) to 90° (UNEFM-PF-502) between lateral shelves of middle nuchal plate. In dorsal view (Figures 6 and 7), neurocranium nearly triangular, tapering anteriorly from posttemporal-supracleithra; nuchal shield broad, with lateral margins of middle nuchal plate distinctly concave; anterior and posterior lateral wings of nuchal shield (= middle plus posterior nuchal plate) approximately equal in size. In ventral view (Figures 6 and 7), parasphenoid weakly elevated relative to dorsal surface of skull. Limits between bones of ventral neurocranium not distinct, vomer almost completely lacking, trigeminofascialis foramen and articulating facet of hyomandibula marked. Intact ornamentation on dorsal surface of skull fine, mostly granular, some forming small striae; no evidence of middorsal groove or furrow on neurocranium or nuchal shield.

Anterior cranial fontanel elliptical (visible only in UNEFM-PF-502), enclosed by mesethmoid (anterior tip) and frontals (medially and posteriorly), not extended posteriorly beyond line across anteriormost portions of sphenotics. Frontals flattened, margins gently concave (almost intact in UNEFM-PF-502, eroded in UNEFM-PF-501). Sphenotic nearly flattened, anteriorly elongate, lateral margin nearly straight, forming an angle of approximately 120° with margin of pterotic. Pterotic large, flattened (UNEFM-PF-501) or slightly convex dorsally (UNEFM-PF-502). Epioccipital large, slightly longer than wide, with six somewhat straight borders. Epioccipital process eroded (based of epioccipital process evident in UNEFM-PF-0415, see 10: Figure 8). Posttemporal-supracleithrum with anterodorsal and posterodorsal wings, latter contacting anterior wing of middle nuchal plate, thus excluding epioccipital from lateral margin of skull. Nuchal shield well developed and broad. Anterior nuchal plate large, longer than wide, suture with parieto-supraoccipital straight; anterolateral margins slightly divergent, posterolateral margins convergent to point posteriorly. Middle nuchal plate butterfly-shaped. Posterior nuchal plate relatively large, with distal margins expanded posteriorly and ventrally into separate wing-like projections (see 10: 172, Figure 8, for description based on UNEFM-PF-0415; new fossils with posterior nuchal plate partially preserved, UNEFM-PF-0501, or completely eroded, UNEFM-PF-502).

#### Discussion

The newly found fossil neurocrania (UNEFM-PF-501, UNEFM-PF-502) are confidently conspecific with those fossil neurocrania (UNEFM-PF-0271, UNEFM-PF-0415) previously described and identified as *Doraops* cf. *zuloagai* by Sabaj Pérez et al. [10]. Less certain is whether all of those fossils, and disassociated fossil pectoral girdles (UNEFM-PF-0278, UNEFM-PF-0413), are conspecific with extant *Doraopszuloagai* , a monotypic genus endemic to the Maracaibo Basin. For example, in the fossil neurocrania, the anterior nuchal plate is relatively long with lateral margins nearly parallel vs. short with strongly oblique, posteriorly divergent lateral margins in extant *Doraops*. To properly identify and place the fossil neurocrania and pectoral girdles in a phylogenetic context, characteristics discernable from the fossils must be coded alongside those for modern representatives of *Doraopszuloagai* , and putatively related genera *Centrodoras*, *Lithodoras*, *Megalodoras* and *Pterodoras* [47]. Such an analysis may reveal the fossils to represent a new lineage within Doradidae, closely related to the clade composed of *Pterodoras* and *Doraops* (sister taxa according to [48]).

† Doras dionae Sabaj Pérez, Aguilera and Lundberg 2007[10]

cf. *Doras* sp. in Aguilera 2004 [45]: 62 (description, biogeography, figure of UNEFM-PF-0411).

Doradidae gen. et sp. indet. 2 in Sánchez-Villagra and Aguilera 2006 [46]: 215 (Table 1), 217 (Figure 2i of UNEFM-PF-0411, correct caption incorrectly refers to Figure 2g, h).

† *Dorasdionae* Sabaj Pérez et al. 2007 [10]: 159–167 (Figure 2) (in part, holotype from Urumaco Formation, El Hatillo, Quebrada Taparito, Falcón State, Venezuela: UNEFM-PF-0411, partial left pectoral girdle with articulated pectoral-fin spine [spine incomplete, approximately distal half missing]). Lundberg et al. 2010 [18]: 293–294 (biogeography, comparisons to extant congeners), 287 (Table 17.2).

#### Description

See Sabaj Pérez et al. [10] for detailed description.

#### Discussion

Fossil material of † *Dorasdionae* is here limited to the holotype (UNEFM-PF-0411). The fossil neurocranium (UNEFM-PF-0477) previously identified as † *Dorasdionae* , is here newly assigned to *Rhinodoras* sp.

Rhinodoras Taphorn and Lilyestrom 1984[22]

*Rhinodoras* sp.
Figure 8 A-F; Table 1

*Dorasdionae* in Sabaj Pérez et al., 2007 [10]: 159–167 (Figure 4) (in part, non-type fossil from Urumaco Formation, Tío Gregorio, Falcón State, Venezuela: UNEFM-PF-0477, partial neurocranium and nuchal shield).

*Rhinodoras* cf. *thomersoni* in Sabaj Pérez et al., 2007 [10]: 174–184 (Figure 13a, b, d, e) (in part, fossil from Urumaco Formation, Tío Gregorio, Falcón State, Venezuela: UNEFM-PF-0478, partial cleithrum including shoulder bulge [complete], anterolateral face, basal portion of dorsal limb [tip missing], and basal portion of postcleithral process [distal portion largely missing]).

#### Newly examined specimens

Neurocranium without anterior ethmoid/vomerine region, UNEFM-PF-503, from Urumaco Formation, Upper Member, Late Miocene. North Corralito, northwestern Venezuela (11°14’44.7″ N, 70°16’26.4″ W).

#### Description

In lateral view, dorsal profile of neurocranium slightly concave from frontals to midpoint of parieto-supraoccipital, and then gently convex from latter point to dorsal spinelet (Figure 8). In transverse plane, dorsal surface gently convex anteriorly across frontals and progressively more arched posteriorly, reaching an angle of approximately 80° between lateral shelves of middle nuchal plate. In dorsal view (Figure 8), true lateral margins of neurocranium and nuchal shield largely eroded in both fossils (i.e., UNEFM-PF-503 and UNEFM-PF 0477), except for shallow concavity corresponding to left lateral margin of middle nuchal plate (UNEFM-PF-0477). In ventral view (Figure 8), parasphenoid weakly elevated relative to dorsal surface of skull, with lateral expansions along its margins; basioccipital prominent (well preserved in UNEFM-PF-503, lacking in UNEFM-PF-0477). Limits between bones of ventral neurocranium not distinct, except for those between prootic, exoccipital and pterotic; vomer completely lacking; trigeminofascialis foramen and articulating facet of hyomandibula marked. First vertebra with lateral expansions directed toward basioccipital; complex vertebrae plus anteriormost free vertebrae deep relative to nuchal shield, with superficial ossification forming a completely closed aortic canal; base of Müllerian rami present but eroded (UNEFM-PF-0477 lacking anteriormost vertebrae). Intact ornamentation on dorsal surface of skull fine, mostly granular, some forming small striae; no evidence of middorsal groove or furrow on neurocranium or nuchal shield in UNEFM-PF-503, but evident at least in part on posterior half of parieto-supraoccipital in UNEFM-PF-0477.

Anterior cranial fontanel elliptical (preserved as its posterior portion between frontals in UNEFM-PF-503), not extended posteriorly beyond line across anteriormost portions of sphenotics. Margins of frontals eroded. Suture between frontal, sphenotic and pterotic obscured by adherent matrix. Sphenotic and pterotic eroded. Epioccipital almost complete, large, slightly longer than wide. Epioccipital process evident and small in UNEFM-PF-502. Posttemporal-supracleithrum lacking, except for posterodorsal wing partially present and contacting middle nuchal plate, thus excluding epioccipital from lateral margin of skull (in UNEFM-PF-503, posttemporal-supracleithrum lacking in UNEFM-PF-0477). Nuchal shield well developed and relatively narrow. Anterior nuchal plate large, approximately as long as wide, suture with parieto-supraoccipital straight; anterolateral margins slightly divergent, posterolateral margins convergent to blunt point posteriorly. Middle nuchal plate butterfly-shaped. Posterior nuchal plate relatively large, with distal margins eroded; suture between posterior and middle nuchal plates transverse relatively to body axis.

#### Discussion

The newly found fossil neurocranium (UNEFM-PF-503) is in all respects consistent with another fossil neurocranium (UNEFM-PF-0477) previously identified as † *Dorasdionae* (e.g. [10]). Those two fossil neurocrania are very difficult to identify due to the preserved nature of the fossils, which lack diagnostic characteristics. Those fossils are confidently treated as conspecific, but less confidently assigned to the modern genus *Rhinodoras* sp *.* *Rhinodorasthomersoni* is endemic to the Maracaibo Basin; however, there are important differences between the fossils and extant *R* *. thomersoni* . In the fossils, the anterior nuchal plate is about as long as wide vs. distinctly shorter than wide in modern *R* *. thomersoni* . Also, in the fossils the superficial suture between the middle nuchal plate and epioccipital is oblique, deflected anterolaterally, and approximately collinear with the suture between anterior and middle nuchal plates. In extant *Rhinodoras* (and *Doras*), the suture between the middle nuchal plate and epioccipital is more transversely aligned, and at an obtuse angle with the suture between the anterior and middle nuchal plates. Another difference involves the suture between the middle and posterior nuchal plates: transversely aligned in fossil (UNEFM-PF-503) vs. oblique, deflected posterolaterally in modern *Rhinodoras* and *Doras*. Furthermore, the parasphenoid in the fossil neurocrania is relatively wider than in extant *Rhinodoras* and *Doras*. Additional fossil material and comparisons to modern *Rhinodoras*, especially adult specimens of *R* *. thomersoni* (currently unavailable), are needed to determine whether the fossil neurocrania treated here are truly referable to *R* *. thomersoni* or representative of an extinct, undescribed doradid.

Doradidae Genus and species indeterminate 1
Figure 9 A-F; Table 1

*Oxydoras* sp. in Aguilera 2004 [44]: 60-61 (description, biogeography, figure of UNEFM-PF-0157).

Doradidae gen. et sp. indet. 1 in Sánchez-Villagra and Aguilera 2006 [46]: 215 (Table 1), 217 (Figure 2d, e of UNEFM-PF-0157, not UNEFM-PF-412 as listed in caption).

**Figure 10:**
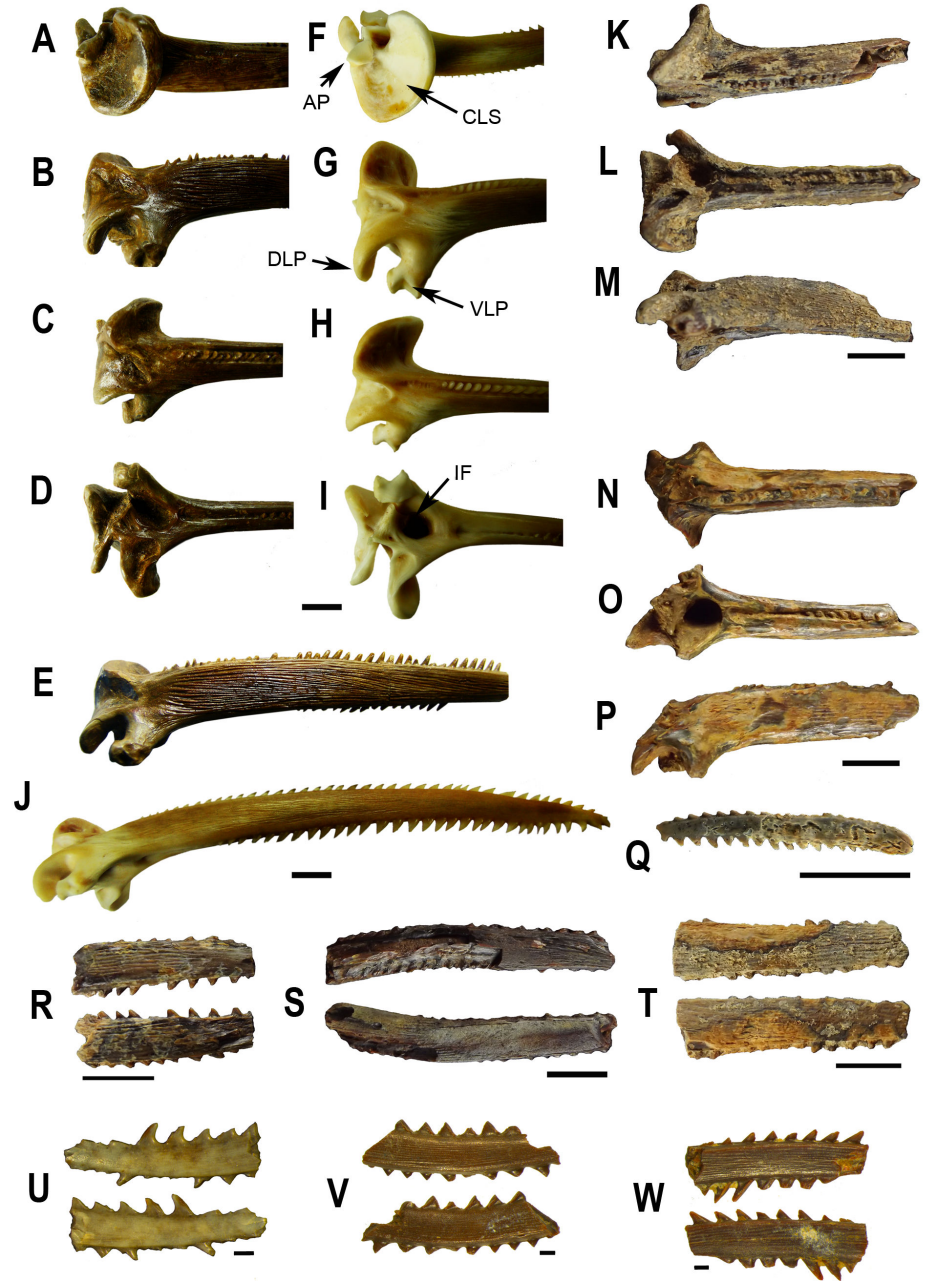
Pectoral spines. **A**-**E**. Fossil Doradidae gen. and sp. ind., STRI- dbid-16241-1/2, from Cocinetas basin, Alta Guajira Peninsula, Colombia, F-J. Recent Doradidae, *Anadoras* sp. MPEG-0062, Santarém, Brazil, K-M. Fossil Doradidae ind., STRI- dbid-13107, **N-P**. STRI- dbid-16241-2/2, Q. STRI- dbid-13114, R. STRI- dbid-390083, S. STRI- dbid-16305; T. STRI- dbid-390020, from Cocinetas basin, Alta Guajira Peninsula, Colombia. **U**. UNEFM-PF-508, V. UNEFM-PF-509, W. UNEFM-PF-510, from the San Gregorio Formation (Vergel Member), Early Pliocene, Northeastern of Urumaco town (11°17’53.9″ N, 70°14’33.7″ W), Venezuela. Scale bar 10 mm from A to T, 1 mm from U to W. Abbreviations: **AP** – axial process; CLS -cleintral surface; **DLP** – dorsal lateral process; **IF** - internal fossa; **VLP** – ventral lateral process.

*Rhinodoras* cf. *thomersoni* in Sabaj Pérez et al., 2007 [10]: 174–184 (Figures 10, 12a) (in part, fossil from Urumaco Formation, Las Huertas, Falcón State, Venezuela: UNEFM-PF-0157, nearly complete neurocranium, nuchal shield, anterior vertebrae and dorsal-fin base; two moieties separated across anterior nuchal plate and epioccipitals).

† *Dorasdionae* in Lundberg et al. 2010 [18]: 293–294 (biogeography, comparisons to extant congeners), 287 (Table 17.2) (in part, neurocranium UNEFM-PF-0157).

#### Description

See Sabaj Pérez et al. [10] for detailed description.

#### Discussion

As its synonymy implies, this particular neurocranium has been exceptionally difficult to identify as congeneric with or closely related to extant doradid taxa. Sabaj Pérez et al. [10] identified the fossil as *Rhinodoras* cf. *thomersoni* based on its dorsal profile and characteristics of the nuchal shield and bones in the otic-temporal region, particularly the sphenotic. The dorsal profile of the fossil skull is incomplete because the neurocranium and nuchal shield are broken into separate moeties across the anterior nuchal plate and epioccipitals. Nevertheless, the intact dorsal profile exhibits a distinct inflection as it begins to rise steeply just anterior to the middle pitline of parieto-supraoccipital, and is level from the suture of anterior and middle nuchal plates to posterior rim of nuchal sheild. This suggests an original condition comparable to modern *R* *. thomersoni* , a species endemic to the Marcaibo Basin. In *R* *. thomersoni* , however, the dorsal profile is strongly oblique, and becomes gradually steeper posteriorly (particularly at middle pitline) before leveling off at about the suture between the anterior and middle nuchal plates. As a result, the dorsal profile of *R* *. thomersoni* has a distinct, rounded, convex hump from the middle pitline to dorsal-fin origin. Sabaj Pérez et al. [10] also noted similarities between the fossil neurocranium and modern *R* *. thomersoni* with respect to the middle nuchal plate. In the fossil, the middle nuchal plate has strongly oblique sides forming a steep triangular transverse arch, and its lateral margins are rounded, deeply concave. Both those conditions also compare well to modern *R* *. thomersoni* .

Sabaj Pérez et al. [10], however, noted that the same features used to assign the fossil to *Rhinodorasthomersoni* are approximated in some species of *Doras* and deep-bodied species of *Trachydoras* (i.e., *T* *. brevis* , *T* *. nattereri* , *T* *. paraguayensis* ). Further reconsideration of the fossil prompted Lundberg et al. [18] to tentatively assign it to the fossil species † *Dorasdionae* .

Based on new observations and comparisons to additional skeletons, we herein consider fossil neurocranium UNEFM-PF-0157 to be unassignable to an extant doradid taxon. A prominent feature of that fossil is the preservation of the ossified transcapular (Baudelot’s) ligaments (see Figure 12a in [10]). The ossified ligaments are not preserved in any of the other fossil neurocrania recovered from the Urumaco Formation. In the fossil UNEFM-PF-0157, the both transcapular processes are preserved; each one exists as a transverse span between the lateral process of basioccipital and exoccipital (medially) and posttemporal-supracleithrum (laterally), with a robust ventral projection slightly deflected posteriorly and subtriangular in posterior view. The opposing ventral medial margins of the paired projections are oblique, forming a broad subtriangular arch with the ventral face of basioccipital as its rounded apex. The lateral margin of each projection is shorter, more vertical, forming a tight rounded arch with the medial wall of the posttemporal-supracleithrum.

Among extant doradids, the transcapular processes are similarly well developed among members of the fimbriate-barbel clade (e.g., *Doras*) and *Rhinodoras* clade (*Orinocodoras*, *Rhinodoras*, *Rhynchodoras*), as well as the genus *Oxydoras* . In the fimbriate-barbel clade, however, the ossified transcapular ligaments are relatively thin, flat and delicate (i.e., unlikely to be preserved in fossil material). Furthermore, fimbriate-barbel doradids typically have a relatively short posttemporal-supracleithrum (vs. elongated in fossil), middle nuchal plate with short anterior wings (vs. long in fossil), posttemporal-supracleithrum isolated from middle nuchal plate (vs. possible short suture between two bones in fossil, difficult to verify because of break), and vomer lacking lateral wings (vs. lateral wings distinct, longer than wide and at 45° angle to shaft in fossil). Consequently, the fossil neurocranium is unlikely to be a member of the extant fimbriate-barbel clade. The robust transcapular processes and the shape of the vomer preserved in the fossil do compare well with those found in extant *Oxydoras* and members of the *Rhinodoras* clade. The fossil, however, does not preserve enough characteristics of the neurocranium to confidently assign it to a particular genus.

Genera and species indeterminate
Figure 10 A-W

#### Newly examined specimens

Three proximal fragments of pectoral spines (STRI-dbid 16241-2/2, STRI-dbid 13107-1), three medial fragments (STRI-dbid 16305, 390020, 390083) and one distal fragment (STRI-dbid 13114) from the Upper Castilletes Formation, Early Pliocene, Cocinetas Basin, Alta Guajira Peninsula (11°51’04.4″ N, 71°19’26.6″ W), Colombia. Three medial fragments of pectoral spines, UNEFM-PF-508 to 510, from the San Gregorio Formation (Vergel Member), Early Pliocene, Northeastern of Urumaco town (11°17’53.9″ N, 70°14’33.7″ W), Venezuela.

#### Description

The cleithral articular surface is semi-circular and wide; axial process pronounced; dorsal-external and ventral-lateral processes well developed. The internal fossa is very large. Pectoral spine shaft gently arched, depressed, subrectangular; surface coarse, ornamented with fine, elongate and parallel striations; dentations along leading edge of spine begin near base and are sharp and gently oriented posteriorly; dentations along trailing edge begin at two times maximal spine width, and are sharp and gently oriented anteriorly. The spine morphotype resembles *Centrochir* and *Platydoras* , sister taxa [48] with *Platydoras* widespread in modern Cis-Andean drainages and *Centrochir* endemic to the modern Magdalena.

## Discussion

The unequivocal association of a diverse fish fauna of Orinoco-Amazon affinities from the Late Cenozoic of Colombia and Venezuela makes a palaeobiogeographic conclusion irrevocable. A river drained its waters in what is today a desert region on the Caribbean coast of easternmost Colombia and northwestern Venezuela. Discoveries of several freshwater forms among the vertebrates of the new fossil sites of Castilletes and the San Gregorio formations are important because they provide a temporal record of an hydrographic connection with the western Amazonian. Given the ages of the localities reported here, the last occurrence of that connection can be temporally situated at approximately between 3.2 to 1.7 Ma.

Today the pimelodid catfish genus *Brachyplatystoma* does not occur west or north of the Andes cordillera or along the coastal ranges of Colombia and Venezuela. An extinct species of *Brachyplatystoma* is represented by † *B* *. promagdalena* from the Middle Miocene La Venta Group in central Colombia [19,41]. Fragmentary remains identified as B. cf. *vaillantii* were collected by Lundberg [41] from the Late Miocene La Venta Formation and co-occurring with † *B* *. promagdalena* . The new fossil records of B. cf. *vaillantii* in the north Guajira Península in Colombia and northwestern Venezuela represent a wider paleogeographic western Amazonian presence of the genus in northern South America.

New discoveries of doradids from the Early Pliocene Castilletes and Late Pliocene San Gregorio formations also represent geochronological evidence for drainage connectivity. Doradids are endemic to South America and distributed in the Magdalena, Maracaibo and cis-Andean basins from the Orinoco to the Rio de La Plata [10,48,49]. One fossil species, † *Dorasdioneae* , and two species assigned here to modern genera *Doraops* and *Rhinodoras* are known from the Late Miocene Urumaco Formation [10]. A fossil collection from Utuquina Valley in Peru, includes *Oxydoras* cf. *niger* and indeterminate doradids. Additional indeterminate doradids were collected from the Middle Miocene La Venta in Colombia [41] and Late Miocene Solimões Formation in Brazil [50]. The new Corralito locality for doradids reported here, together with the Tío Gregorio locality, represent the top of the Urumaco Formation section. Those Corralito and Tío Gregorio localities are separated laterally ca. 4 Km East-West from each other by a complex fault system [51,52].

## Changes in Palaeohydrographic Patterns and the Fossil Record of Fishes

The Andes of Colombia consist of three nearly parallel and north-south oriented mountain ranges (Eastern Central and Western cordillera) that merge into a single one near the Ecuadorian border. Between these ranges lie two river valleys, the high and narrow Cauca valley to the west, and the low and broad Magdalena valley to the east. The Magdalena River is the largest fluvial system in Colombia (1,612 km long) and originates from headwaters in the Andean Cordillera (elevation of 3,300 m) [53]. There is a rich vertebrate fauna of Middle Miocene La Venta fossils from the upper Magdalena Valley (Honda Group [16]:) and knowledge of the ancient Amazonian freshwater fish fauna is greatly expanding [18]. This fauna includes a lungfish ( *Lepidosiren* ), river rays ( *Potamotrygon* *idae* ), *Arapaima* , various characiforms, a cichlid, and catfishes in the families Callichthyidae, Loricariidae, Doradidae and Pimelodidae ( *Phractocephalus* , cf. *Pimelodus* , † *Brachyplatystoma* *promagdalena* Lundberg 2005 [19], and B. cf. *vaillantii* Valenciennes 1840 [18,20]. Downstream in the lower Magdalena River, no Miocene or Pliocene fossiliferous fluvio-lacustrine deposits are found. Upstream deposits in the Magdalena Valley between the Eastern and Western Cordilleras of Colombia indicate a Late Miocene age for the Magdalena River [54]. The onshore plains in the current Magdalena delta are covered by thick Pliocene-Quaternary terrigeneous deposit that resulted from successive migrations of the Magdalena River [55]. During the Pliocene, the mouth of the Magdalena River was situated near the town of Galerazamba; it migrated westward during the Pleistocene towards the city of Cartagena, and then migrated to the north in the Recent [56]. A paleohydrographic hypothesis that requires testing is the Pliocene/Pleistocene persistence of the Magdalena river drainage throughout the Cesar Valley, between the Santa Marta Massif and the Sierra de Perija to the Alta Guajira Peninsula.

Presence of the Misoa delta in the Middle Eocene of the Maracaibo Basin, northwestern Venezuela, has been suggested as evidence for a large river system, flowing in a south-north direction and draining the Central Cordillera of Colombia and the Guyana Highlands [2]. The late Middle Eocene uplifting of Western Venezuela changed the hydrographic setting, and a new region of delta-building shifted to the south, represented by the extensive Carbonera Formation of Late Eocene to Oligocene age in the Llanos Basin of Colombia and southwestern Venezuela. In the earliest Miocene, the western margin of the Falcón Basin, situated to the east of the Maracaibo Basin, suggests the presence of a river with clear faunal affinities to the Amazon and Orinoco. In any case, from the mid Early Miocene until the end of the Middle Miocene, a fluvial-deltaic sequence called the proto-Orinoco was deposited in the northwestern Falcón Basin. The deformation and uplift of the Eastern Cordillera of Colombia and of the southwestern end of the Mérida Andes caused in the late Middle Miocene deflected the distal course of the river to a west-east direction [2].

The age of initial orogenic uplift of the Mérida Andes has been suggested to be Miocene [57]. However, according to Higgs [58] the Mérida Andes uplift started near 5 Ma, and simultaneously that of the Perijá, Santa Marta, Lara-Falcón and Guajira [59,60]. Based on our newly found paleontological evidence, we are in agreement with Higgs [58] that the paleofluvial system of the Amazonian-Orinoco switched to an eastward course approximately 2.5 Ma, not nearly as long ago as 12 Ma. Alternatively, the fish faunas we are describing from Castilletes and Urumaco are the last remnants of western Amazonian faunas currently represented, in part, in the Maracaibo basin and the Magdalena River.

The Amazon basin has changed its extent and orientation since the Late Cretaceous [54]. During the Upper Miocene and Pliocene, the Purus Arch in Central Amazonia played a major role as a geographic barrier between the Solimões (upper) and Amazonas (lower) basins. From the Pliocene to Pleistocene, the Purus arch underwent subsidence and the wide connection between the two basins was established [61]. Plate tectonics and mantle convection produced a tilt of northern South America to the East, resulting in the eastward drainage of the Amazon by the late Neogene, at rates and times which are being modeled [62]. Temporal estimates for the establishment of the transcontinental Amazon fluvial system range widely from Late Miocene [7,54,63-68] to Late Pleistocene [69,70]. Figueiredo et al. [68] suggested that the transcontinental river initiated between 11.8 and 11.3 Ma ago (Middle to Late Miocene) and reached its present shape and size during the late Pliocene. The age of initial orogenic uplift of the Mérida Andes has been suggested to be Miocene [57]. However, according to Higgs [58] the Mérida Andes uplift started near 5 Ma, and was simultaneous to that of the Perijá, Santa Marta, Lara-Falcón and Guajira [59,60]. However, Albert et al. [71] concluded that the minimum age for the isolation of the western Amazon and Orinoco basins is 9 Ma, and the minimum date for the isolation of the modern Maracaibo and Orinoco basins in 8 Ma. Ultimately, well dated biological events based on molecular phylogenetics [72] and well-dated fossils from several basins [73] will provide tests for the geographic and temporal hypotheses generated by geological studies.

Further discoveries could support the hypothesis that the fossil catfishes from the ages discussed here for the Castilletes and Urumaco deposits have primary affinities with the fauna found today in the Magdalena River and the Maracaibo basin. This would mean that they existed in a relictual and peripheral river system that was cut off earlier from the Amazon-Orinoco. Whatever the case, the palaeontological discoveries of freshwater forms in the northern Neotropics are playing a key role in testing and providing geochronological information for solving the region’s past biogreography.
